# Supplementary material for: Fenestrated Physician-Modified Endografts for Preservation of Main and Accessory Renal Arteries in Juxtarenal Aortic Aneurysms
Source: J Clin Med. 2023 Jul 15;12(14):4708. doi: 10.3390/jcm12144708 (PMC10416159; doi:10.3390/jcm12144708)
Supplement: Supplementary file 1 [file jcm-12-04708-s001.zip › jcm-2440083-supplementary.pdf]

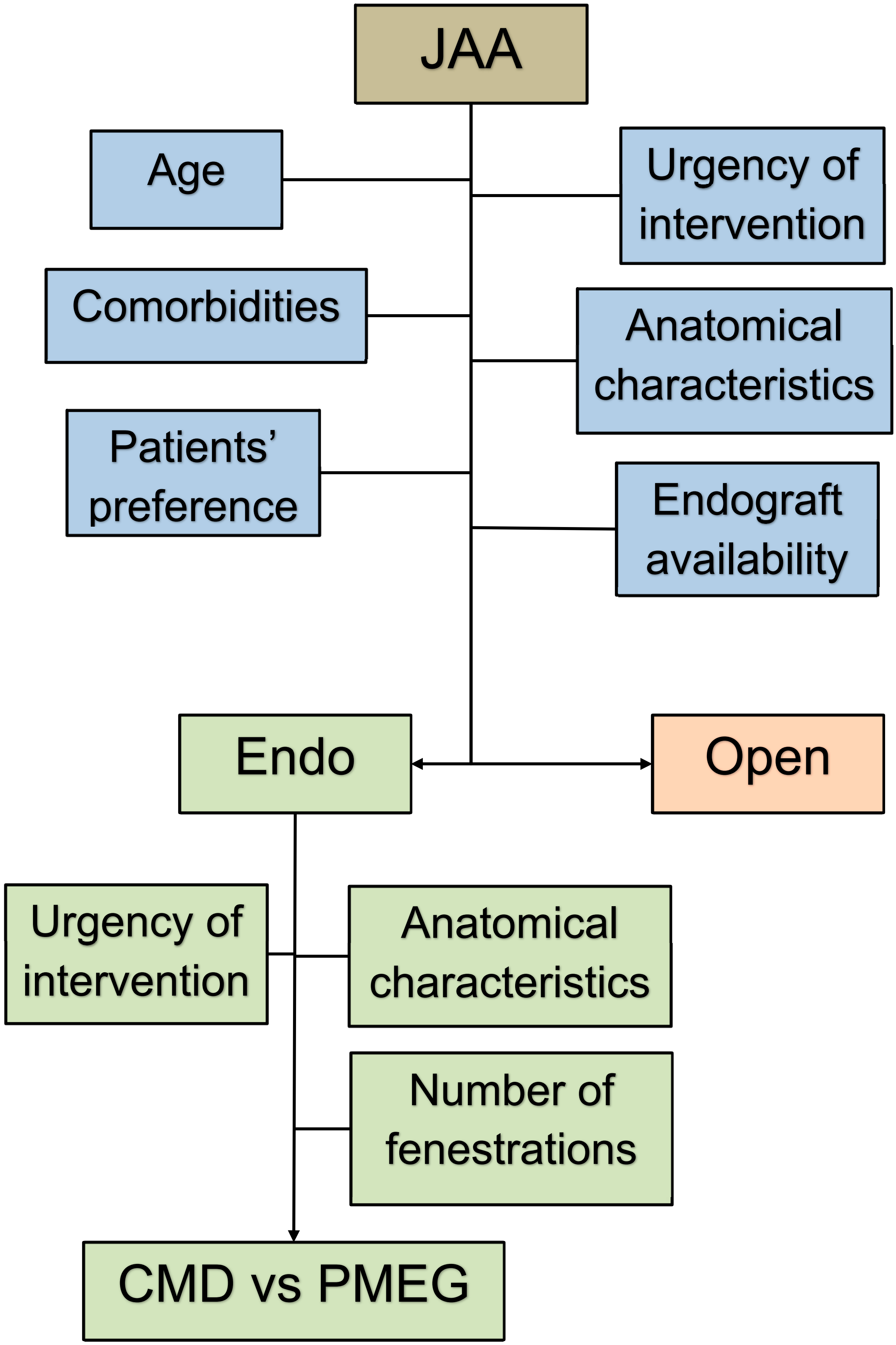

Figure S1. Flowchart of factors influencing treatment decision for patients with juxtarenal aortic aneurysms in our institution. Open repair with suprarenal clamping was offered to all patients fit for open surgery

(functional capacity ≥ 4 METs). Custom-made fenestrated devices were preferred in patients less fit for open repair and where the procedure was planned 3-4 months in advance, with difficult target vessel anatomy and/or > 2 target vessels. Physician-modified devices were considered in all patients with ruptured or symptomatic juxtarenal AAA as well as in elective cases with 1-2 target vessels". Custom-made devices (CMD) and physician-modified endograft (PMEG)

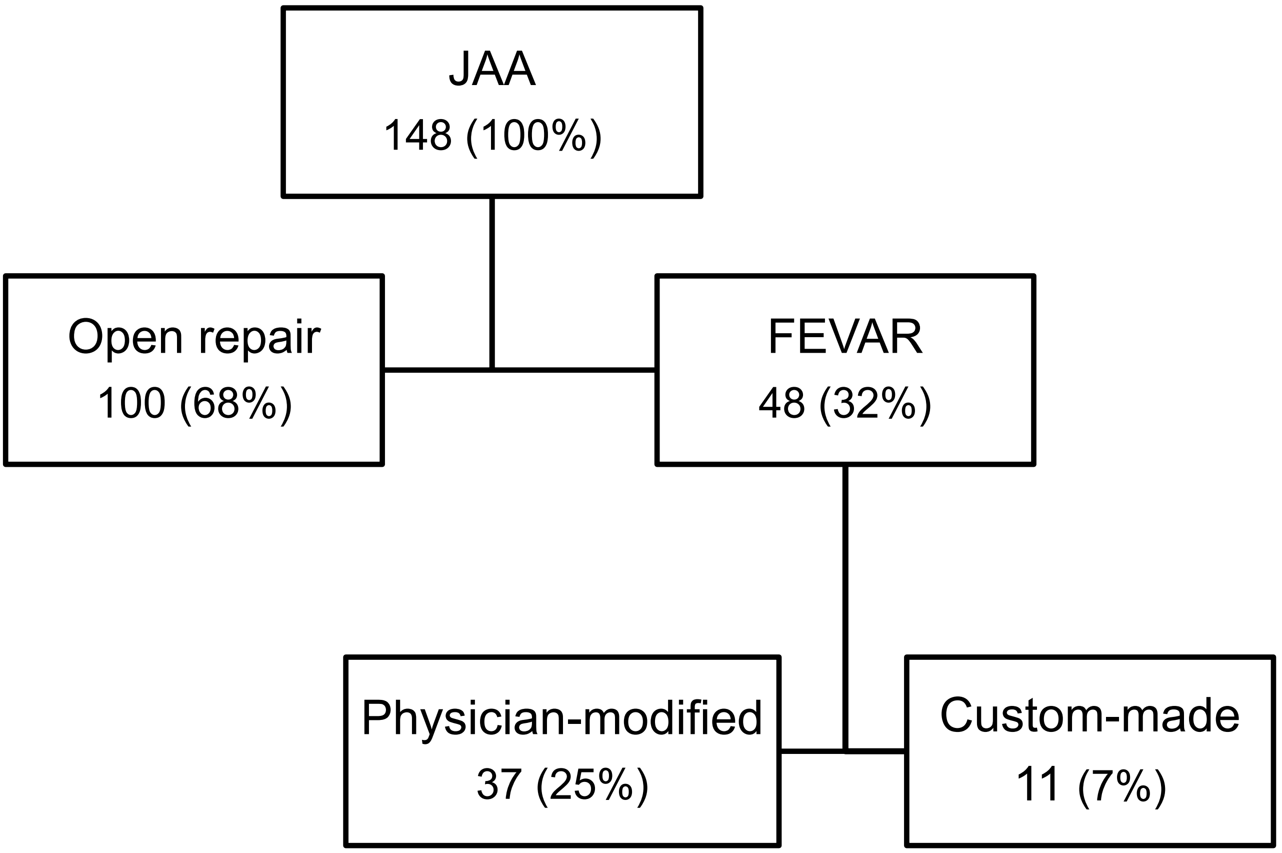

Figure S2. Flowchart of all patients with juxtarenal aortic aneurysms (JAA) treated between March 2019 and January 2023 in our department. Fenestrated endovascular aortic repair (FEVAR)

Figure S3. Failed catheterization of an accessory renal artery (ARA), which remained patent without bridging stent during follow-up. A: intraoperative angiogram prior deployment of physician-modified stent-graft with 1x fenestration for the ARA; B: final angiogram intraoperatively with patent ARA; C: one-year postoperative CT-angiography with patent ARA and no endoleak

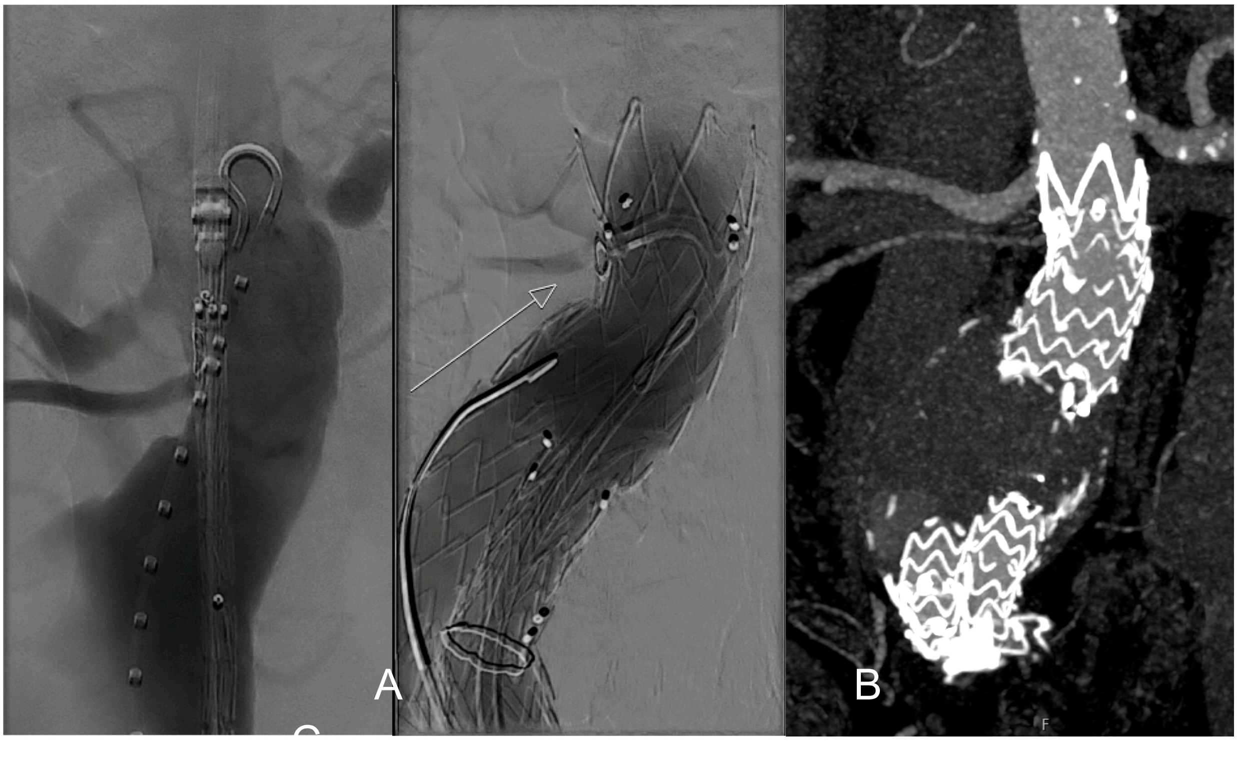

Patient number
Gender
Type of graft
Date of Admission
Date of Surgery
Date of discharge
Date of Death
Cause of Death
Life Status on Study Closing Date
**Patient characteristics**
Height [m]
Weight [kg]
BMI [kg/m2]
Coronary artery disease
Atrial Fibrillation
Myocardial Infarction
Arrhythmia
Unstable Angina
Heart insufficiency
CABG
PTCA/Stent
Hypertension
Hypercholesterolemia
Smoker
Active Smoking
COPD
Steroids/Home O2
Diabetes Mellitus
Renal Impairment
Baseline Creatinine [ul/L]
Baseline GFR [ml/min]
Stroke
TIA
Connective Tissue Disorder
Cancer
Peripheral Arterial Disease
SVS Cardiac Score
SVS Respiratory Score
SVS Renal Score
SVS Hypertension Score
SVS Age Score
SVS Total Score
ASA Clinical Score

**Preoperative Medication**
Aspirin
Plavix
NOAK/OAK
Anticholesterol Medication
Nitrates
Ca Channel Blockers
ACE Inhibitors / AT Receptor Inhibitor
B-Blockers
**Aneurysm characteristics**
Previous aortic operation
Kind of previous operation
Previous abdominal surgery
CTA Preoperative
CTA Date
Type of Aortic Pathology
Largest Diameter of Aneurysm [mm]
Status of Aneurysm
Organ Complication
Type of Aneurysm
Proximal Endograft Size
Proximal Aortic Diameter
Proximal Oversize
Alpha Angle
Beta Angle
Calcification in Proximal Landing Zone
Thrombus in Proximal Landing Zone
Target vessels
Diameter of Target Vessel
Technical Success
Access Vessel
Steerable Sheath
Total Operation Time [min]
Type of Contrast
Volume of Contrast [ml]
Dosis Area Product (DAP) [mGy/cm2]
Fluoroscopy Time [min]
Endoleak at Final Angiography
Type of Endoleak
Immediate Intervention
Blood Loss [ml]
EK [units]
FFP [units]

TC [units]
Cellsaver [ml]
Hospital Death
**Complications**
SIRS/Sepsis
Myocardial Infarction
Respiratory Failure
Stroke/TIA
Paraplegia
Renal failure
Arrhythmia
Pancreatitis
Mesenteric ischemia
Ischemic colitis
Other GI Complications
Bleeding
Early Re-Intervention
Access Complication
Creatinine at Discharge [ul/L]
GFR at Discharge [ml/min]
ICU Length of Stay
IMC Length of Stay
CTA Before Discharge
Date of CTA at Discharge
Endoleak
Type of Endoleak
Intervention
**Follow-up**
Date of Follow Up 1
Date of CTA
Any Complication
Any Secondary Intervention
Date of Secondary Intervention
Kind of Secondary Intervention
Any Branch Stenosis/Occlusion
Any Aneurysm Rupture
Sac Change
Final Sac diameter
Any Late Endoleak
Type of Endoleak

Table S1. Collected study parameters
